# Progress in Understanding Ferroptosis and Its Targeting for Therapeutic Benefits in Traumatic Brain and Spinal Cord Injuries

**DOI:** 10.3389/fcell.2021.705786

**Published:** 2021-08-04

**Authors:** Xinli Hu, Yu Xu, Hui Xu, Chenqiang Jin, Haojie Zhang, Haohan Su, Yao Li, Kailiang Zhou, Wenfei Ni

**Affiliations:** ^1^Department of Orthopaedics, The Second Affiliated Hospital and Yuying Children’s Hospital of Wenzhou Medical University, Wenzhou, China; ^2^Zhejiang Provincial Key Laboratory of Orthopaedics, Wenzhou, China

**Keywords:** ferroptosis, programmed cell death, CNS trauma, spinal cord injury, traumatic brain injury

## Abstract

Acute central nervous system (CNS) trauma, including spinal cord injury (SCI) and traumatic brain injury (TBI), always leads to severe sensory, motor and autonomic nervous system dysfunction due to a series of processes, including cell death, oxidative stress, inflammation, and excitotoxicity. In recent years, ferroptosis was reported to be a type of programmed cell death characterized by the consumption of polyunsaturated fatty acids and the accumulation of membrane lipid peroxides. The processes that induce ferroptosis include iron overload, imbalanced glutathione metabolism and lipid peroxidation. Several studies have indicated a novel association of ferroptosis and acute CNS trauma. The present paper reviews recent studies of the occurrence of ferroptosis, stressing the definition and process of ferroptosis and metabolic pathways related to ferroptosis. Furthermore, a summary of the existing knowledge of the role of ferroptosis in CNS trauma is presented. The aim here is to effectively understand the mechanisms underlying the occurrence of ferroptosis, as well as the relevant effect on the pathophysiological process of CNS trauma, to present a novel perspective and frame of reference for subsequent investigations.

## Introduction

Acute central nervous system (CNS) trauma, including spinal cord injury (SCI) and traumatic brain injury (TBI), is a primary cause of mortality and morbidity worldwide (Hu et al., 2020). Each year, approximately 50 million individuals worldwide suffer from TBI (Maas et al., 2017). SCI, another form of neurological trauma, has an incidence of over 2.5 million people globally (Le Corre et al., 2018). Regarding the mechanisms associated with acute CNS trauma, existing studies have reported the death of neuronal cells as a vital pathological event in CNS trauma (Shi et al., 2021). The pathophysiology of CNS trauma includes primary injury and secondary injury (Ng and Lee, 2019). Secondary damage mainly includes the release of intracellular and intra-axonal contents, such as glutamate, reactive oxygen species (ROS), and Fe^2+^ (Hu et al., 2020). Glutamate-induced excitotoxicity is one of the major events in secondary CNS trauma. Excitotoxicity leads to mitochondrial damage and dysfunction, increased ROS release and DNA damage, all of which lead to different types of cell death (Hiebert et al., 2015). Therefore, the ability to control the various cascades of injury-induced cell death during secondary injury is a vital problem that must be solved to develop effective therapies that promote recovery after CNS trauma. Rapidly accumulating evidence indicates that neurons are vulnerable to ferroptotic cell death in response to CNS trauma (Geng et al., 2021).

Compared with other forms of programmed cell death, such as autophagy, pyroptosis and necroptosis, ferroptosis exhibits special morphological and biological features. The morphological characteristics of ferroptosis include the rupture of cell membranes, but chromatin condensation is lacking in the cell nucleus (Čepelak et al., 2020). Using electron microscopy, ferroptosis has been shown to trigger cell death by inducing a loss of mitochondrial cristae, increased density of mitochondrial membrane, and shrunken mitochondria (Neitemeier et al., 2017). The biological characteristics of ferroptosis include the accumulation of ROS and iron. This characteristic is not consistent with autophagy and apoptosis. Although the mechanisms of ferroptosis have been well studied, the possible molecular and pathogenic mechanisms involved in CNS trauma remain unclear, indicating that the elucidation of the mechanisms of ferroptosis is important. This review aims to summarize recently conducted studies on CNS trauma and ferroptosis to determine how ferroptosis modulates CNS trauma.

## Definition and Process of Ferroptosis

The term ferroptosis emerged in 2012 to describe the form of cell death triggered by the small molecule erastin, thereby inhibiting cystine import, triggering the inactivation of phospholipid peroxidase glutathione peroxidase 4 (GPX4) and consumption of glutathione (Dixon et al., 2012). GPX4 is capable of converting lipid hydroperoxides (L-OOH) with toxic potential into non-toxic lipid alcohols (L-OH) (Forcina and Dixon, 2019). The inactivation of GPX4 mediated by erastin-induced glutathione (GSH) consumption or treatment with the direct GPX4 inhibitor (1S,3R)-RSL3 (RSL3) leads to preponderant lipid peroxidation, which causes cell death. Ferroptosis was suggested to be involved in pathological processes after CNS trauma (Xie et al., 2019). First, the acute phase of CNS trauma is characterized by immediate hemorrhage (Fletcher-Sandersjöö et al., 2021). In addition, hemoglobin (Hb), the most abundant protein in blood, is metabolized into ferrous/ferric iron, which causes iron overload at the injury site (Wan et al., 2019). Fe^3+^ is introduced in the cell through the membrane protein transferrin receptor 1 (TFR1) and subsequently localizes to endosomes (Forcina and Dixon, 2019). Iron reductase is specifically located in endosomes and reduces Fe^3+^ into Fe^2+^ (Stockwell et al., 2017). Last, divalent metal transporter 1 (DMT1) transports Fe^2+^ to the labile iron pool (LIP) in unstable iron pools within the cytoplasm, and large amount of iron is stored within ferritin, a protein complex responsible for iron storage (Harrison and Arosio, 1996). However, under trauma conditions, the LIP, the chemically reactive and kinetically labile pool, releases Fe^2+^ into the cytoplasm (Ke and Qian, 2007). Excess iron is capable of synthesizing hydroxyl radicals with high oxidizing activities (such as ROS) through the Fenton reaction, in which Fe^2+^ reacts with hydrogen (Forcina and Dixon, 2019). This process leads to ferroptosis. At the same time, oxidation of polyunsaturated fatty acid (PUFA)-containing phospholipids (PLs) the plasma membrane was recently identified as a key process for ferroptosis (Stockwell et al., 2017). Acyl-CoA synthetase long-chain family member 4 (ACSL4) preferentially activates PUFA free fatty acids to generate PUFA-CoAs (Stockwell et al., 2017). Lysophosphatidylcholine acyl transferase 3 (LPCAT3) insert acyl-CoA molecules into lysophosphatidylcholine to synthesize phosphatidylcholine (Doll and Conrad, 2017). Generally, these 2 enzymes are conducive to defining a lipid metabolic pathway critical for inserting PUFAs into membrane PLs and improving susceptibility to ferroptosis (Doll et al., 2017). In addition, ferroptosis is regulated by the GPX4-GSH-cysteine axis. In this axis, vital processes comprise cystine uptake through system Xc^–^, reducing the processing of phospholipid hydroperoxides into lipid alcohols by GPX4, GSH biosynthesis, and reducing the transformation of cystine into cysteine (Chen J. et al., 2020). This procedure can relieve ferroptosis.

## Metabolic Pathways Related to Ferroptosis

**FIGURE 1:**
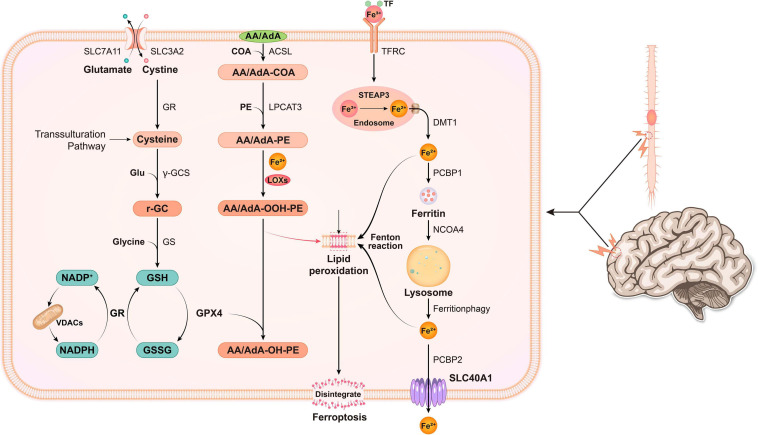
In nerve cells, various pathogenic factors cause abnormal increases in Fe^3+^ levels, and Fe^3+^ is then converted into Fe^2+^ by STEAP3. Free iron (Fe^2+^) is transported from the endosome by DMT1. Some of the free iron is stored in ferritin through PCBP1, and some is transported out of the cell through SLC40A1. Free iron is released upon ferritin degradation *via* the ferritinophagy pathway, which is mediated by NCOA4, and might cause lipid ROS. System xc- is a cystine/glutamate antiporter. Intracellular cystine is reduced to cysteine for the biosynthesis of GSH. When cystine is inside the cell, cystine is reduced to cysteine, and the level of cysteine is supplemented by the transsulfuration pathway. Subsequently, cysteine is used for the biosynthesis of GSH. GPX4 uses two GSH molecules as electron donors to reduce phospholipid hydroperoxides (PL-OOH) to the corresponding alcohols, leaving GSSG (oxidized GSH) as a by-product. Then, GSSG is recycled by GSH reductase in an NADPH-dependent manner. The ion selectivity of VDAC determines whether cations or anions enter the mitochondria, thus affecting the oxidation state of NADH. ACSL4 is required to activate PUFAs, especially AA and AdA, to AA-CoA, and AdA acyl-CoA derivatives. These derivatives are esterified by LPCA T3 into AA-PE and AdA-PE, which are then catalyzed by the iron-containing enzyme lipoxygenase (LOX) to generate fatty acid hydroperoxides, thus leading to ferroptosis.

Three metabolic pathways have been shown to be closely associated with the regulation of ferroptosis, including glutathione metabolism, lipid metabolism and iron metabolism (Shen et al., 2020). Thus, we will discuss the relationship between the three metabolic pathways and ferroptosis below. A summary of the metabolic pathways related to ferroptosis is shown in Figure 1.

### Glutathione Metabolism Is Related to Ferroptosis

System Xc^–^ is a Na^+^-dependent cysteine-glutamate antiporter located in the membrane that is composed of SLC3A2 and SLC7A11 (xCT) and responsible for the exchange of intracellular glutamate for extracellular cystine at a 1:1 ratio (Shen et al., 2020). If transported in the cell, cystine is reduced to cysteine *via* the GSH synthesis process (Čepelak et al., 2020). Among the 3 amino acids identified in GSH, cysteine is present at the lowest abundance, and thus it is recognized as the rate-limiting residue to achieve *de novo* GSH synthesis (Dixon et al., 2014). Cysteine is synthesized from methionine in several cell types through transsulfuration. The cysteine-glutathione channel was suggested to be a crucial upstream signaling pathway regulating ferroptosis (Dixon et al., 2014). System Xc^–^, which is not regulated by ATP, is activated by high concentrations of intracellular glutamate. Thus, it is particularly sensitive to the extracellular glutamate concentration. Millimolar concentrations of extracellular glutamate block system Xc^–^ activity and trigger oxidation-related cell death, which was previously considered oxytosis (Tobaben et al., 2011). Increased extracellular glutamate levels occurring following numerous types of brain trauma suppress system Xc^–^ transport, thereby inducing ferroptosis (Latunde-Dada, 2017). The direct inhibition of system Xc^–^ was recently reported to induce cell death by decreasing cysteine uptake, thereby causing GSH depletion and impairing the ability of the cell to resist oxidation-related stress (Gout et al., 2001). Moreover, several studies have shown that GSH depletion induced by buthionine sulfoximine (BSO), diverse pharmacological inhibitor-2 (DPI2), an active metabolite of acetaminophen, and N-acetyl-p-benzoquinone imine (NAPQI) is associated with ferroptosis. Glutathione peroxidase 4 (GPX4) is a member of the GPX family that is involved in ferroptosis. GPX4, together with GSH, reduce organic peroxide (ROOH) or free hydrogen peroxide (H_2_O_2_) to the corresponding alcohols and water. Additionally, the catalytic cycle of GPX4 consumes 2 GSH molecules to generate oxidized glutathione (GSSG) (Wu et al., 2019). GSSG can be recycled as GSH through GSH reductase in an NADPH-dependent manner (Farina and Aschner, 2019). L-OOH formation and L-OOH reduction into L-OH are balanced constantly by GPX4 (Li et al., 2021). L-OOH perform oxidation using Fe^2+^ to yield significantly reactive alkoxy radicals (L-O-) (Shen et al., 2020). The mentioned radicals directly damage nearby PUFAs *via* a free radical-mediated chain reaction process (Xie et al., 2016). Following the loss or inactivation of GPX4, L-OOH accumulates in larger concentrations than normal, thereby causing more significant L-O- production and possibly resulting in catastrophic membrane injury (Wu et al., 2019).

### Iron Metabolism Is Related to Ferroptosis

Iron is a vital nutrient to maintain physiological function. Iron has two states in humans, free and bound iron. The majority of bound iron is in the form of ferritin, for example, iron-sulfur nanoclusters and hemoglobin, whereas free iron, or non-bound iron, is mainly present inside non-iron-sulfur nanoclusters or haem (Chen Y. et al., 2020). Free iron consists of ferric iron and ferrous iron. Although researchers have not clearly determined how iron affects the lipid oxidation process to promote ferroptosis, iron is an essential component of oxidative cell death (Tang et al., 2021). Ferroportin, ferritin, transferrin and other proteins that contain iron for iron degradation, utilization, storage, and uptake help facilitate ferroptosis (Cotticelli et al., 2019). A large amount of free Fe^3+^ in the blood forms a complex composed of extracellular transferrin (Tf) and transferrin receptor 1 (TFR1) in transplanted cells and the cell membrane during endocytosis (Biasiotto et al., 2019). Next, based on the acidic pH environment in the endosome, Fe^3+^ is degraded into the highly reactive Fe^2+^ by the six-transmembrane protein prostate 3 (STEAP3) (Shen et al., 2020). Through a process mediated by divalent metal transporter 1 (DMT1, also known as SLC11A2), Fe^2+^ translocates from endosomes into the cytoplasm and subsequently forms an iron pool that is not stable inside the cytoplasm (Ke and Qian, 2007). Fe^2+^ inside the iron pool undergoes partial delivery to ferritin by directly binding to Poly(rC)-binding protein 1 (PCBP1) to protect cells and tissues from iron-induced injury, whereas some Fe^2+^ also binds to PCBP2 and then is removed from the cell *via* SLC40A1 located on the cell membrane (Tang et al., 2021). In a normal environment, the concentration of intracellular iron remains steady. If the balance is disrupted, such as iron under overload, excessive iron and divalent ferrous ion Fe^2+^ are precisely capable of reacting with ROOH or H_2_O_2_ to yield soluble lipid alkoxy (RO–) or hydroxyl (HO–) radicals, respectively (Tang et al., 2021). These substances are the source of major ROS generated from Fe^2+^ inside cells through a process known as the Fenton reaction (Dixon and Stockwell, 2014). They easily acquire electrons from other molecules to facilitate lipid peroxide formation and induce ferroptosis.

### Lipid Metabolism Is Associated With Ferroptosis

The accumulation of lipid peroxidation is suggested to be critical in the execution phase, with PUFAs being vital (Doll and Conrad, 2017). New long-chain fatty acids are converted to CoA to be incorporated into phospholipids (PLs) (Liang et al., 2019). A vital enzyme regulating ferroptosis, ACSL4, catalyzes arachidonic acid (AA) and adrenic acid (AdA) acylation reactions (Doll et al., 2017). Upon catalysis by LPCTA3, the acetylation product is combined with phosphatidylethanolamines (PE) in the membrane PL to produce PE-AdA and PE-AA (Kagan et al., 2017). After AA/AdA-PE is generated, activated lipoxygenase (LOXs), especially 15-lipoxygenase (15-LOX), catalyze the transformation of AA/AdA-PE into the proferroptotic lipid peroxidation product AA/AdA-OOH-PE, which leads to lipid injury that is impossible to repair and a permeabilized membrane (Doll and Conrad, 2017). In addition, some studies suggest that the function of the LOXs family of enzymes depends on iron (Magtanong and Dixon, 2018; Wu et al., 2019). However, at present, no conclusive evidence for the iron dependence of LOX is available, which deserves further study. The method of eliminating AA/AdA-OOH-PE only depends on GPX4, and this enzyme exhibits the ability to reduce toxic, membranous lipid hydroperoxides into non-toxic lipid alcohols (Yang et al., 2014). Thus, the GPX4 inactivation process promotes the accumulation of lipid peroxidation and ferroptosis initiation.

## The Effect of Ferroptosis on TBI and SCI

When external forces are applied to the brain and spinal cord, blood vessels rupture, the blood-brain barrier and blood-spinal cord barrier are destroyed, and blood leakage occurs inside the brain and spinal cord (Wan et al., 2019). Accordingly, iron from hemoglobin present in the blood is released and induces iron overload (Shen et al., 2020). Divalent ferrous ion Fe^2+^ produces excess ROS through the Fenton reaction. The major effect exerted by ROS is oxidative injury to biofilms containing PUFAs (Xie et al., 2016). Meanwhile, cholesterol and PUFAs are abundant in neuronal membranes and exhibit high susceptibility to oxidizing processes induced by ROS (Shichiri, 2014). Moreover, the capability of neurons to scavenge ROS through an autonomous approach is limited (Hiebert et al., 2015). Thus, neurons exhibit exceptional vulnerability to iron overload. TBI and SCI share many of the same pathophysiological features. The present review investigates the mechanisms by which iron and ferroptosis affect TBI and SCI to determine the effects of iron metabolic processes on TBI and SCI and to identify a novel and feasible treatment for SCI and TBI targeting ferroptosis and iron (Table 1).

**TABLE 1:** Ferroptosis-associated drugs used to treat TBI and SCI.

**Disease**	**Drug**	**Target**	**Potential mechanism**
SCI	Deferoxamine	Iron chelator	Increased xCT, GSH, and GPX4 levels
	SRS 16–86	Small-molecule, ferroptosis-specific inhibitor	Upregulated GPX4, GSH, and xCT levels
	Proanthocyanidin	Lipid peroxidation inhibitor	Upregulated GPX4, GSH, HO-1, and xCT levels
	Liproxstatin-1	Lipid peroxidation inhibitor	Suppresses mitochondrial lipid peroxidation, reduces MDA levels, and upregulates GSH, GPX4, and FSP1
TBI	Baicalein	12/15-lipoxygenase inhibitor	Suppressed the 15-lipoxygenase pathway
	miR-212-5p agomir	PTGS2 inhibition	Suppressed the enzyme catalyzing lipid oxidation
	Ferrostatin-1	Lipid peroxidation inhibitor	Reduces iron deposition

### The Effect of Ferroptosis on SCI

After spinal cord injury, the spinal cord exhibits substantial hemorrhaging, erythrocyte aggregation, cell rupture, and hemolysis and local iron overload (Derry et al., 2020). Moreover, stress induces the production of a large amount of ROS and increases the excitotoxicity of glutamate (Kang et al., 2020). All of these factors induce ferroptosis. Ferroptosis signaling critically affect SCI. Mitochondrial morphology is a vital criterion that supports a role for ferroptosis in SCI. Using transmission electron microscopy (TEM), Yao et al. revealed variations within the mitochondria of ferroptotic cells (Yao et al., 2019). They also observed noticeable changes in the levels of ferroptosis markers such as GPX4, xCT, and glutathione in spinal cord tissues from rats with SCI. In addition, using real-time polymerase chain reaction, they detected increased mRNA levels of ferroptosis-related genes, such as iron-responsive element-binding protein 2 (IREB2) and acyl-CoA synthetase family member 2 (ACSF2). As suggested by the results described here, ferroptosis critically affects SCI. Reactive astrocytes, which are the major component of the glial scar and function as a chemical and physical barrier to axon regeneration, are associated with the failure of axon regeneration in individuals with SCI (Patil et al., 2021). Deferoxamine (DFO), an iron chelator, is capable of suppressing ferroptosis triggered by the increase in the activity of the Xc^–^/GPX4 axis in SCI, and the antigliotic effect of DFO was consistent with its effect on GPX4 upregulation (Derry et al., 2020). This result indicated a potential link between gliosis and ferroptosis following SCI, which requires further research. Subsequently, ferroptosis has been extensively studied in spinal cord injury. Zhang et al. (2019) investigated the noticeable effect of the ferroptosis inhibitor SRS 16-86 on attenuating ferroptosis in an animal model of SCI. According to the authors of this study, more common mitochondrial morphological characteristics were observed upon SRS 16-86 treatment at 24 h, indicating a greater volume and larger mitochondrial cristae in contrast to the SCI-vehicle cohort. Regarding the mechanisms by which SRS 16-86 regulates ferroptosis, this study has also illustrated that SRS 16-86 suppresses ferroptosis by upregulating xCT, GPX4, and GSH and suppressing lipid peroxidation. Furthermore, the authors found that SRS 16-86 treatment reduced astrogliosis and increased neuronal survival after SCI. Nuclear factor-erythroid 2-related factor 2 (Nrf2), a master regulator of the cellular antioxidant response, involves in regulating dozens of genes which related to ferroptosis, such as GPX4 and xCT (Abdalkader et al., 2018). Additionally, oxygenase-1 (HO-1) plays a critical role in heme metabolism which have protective or detrimental effects during ferroptosis (Chiang et al., 2018). In addition, thiobarbituric acid reactive substances (TBARS) is commonly quantified to assess the extent of lipid peroxidation (Zhou et al., 2020). Zhou et al. (2020) investigated another ferroptosis inhibitor, proanthocyanidin (PAC), which attenuates ferroptosis through different mechanisms after SCI. According to their results, PAC treatment noticeably reduced the levels of iron, ALOX15B, ACSL4, and TBARS and increased heme HO-1, Nrf2, GPX4, and GSH levels in SCI models. These results reveal the protective effects of the PAC treatment on ferroptosis through iron chelation and antioxidant activity. LPCAT3 and ACSL4 contribute to cellular lipid generation and essentially modulate ferroptosis (Yuan et al., 2016). PAC treatment insignificantly altered the increase in LPCAT3 levels inside the injured spinal cord, but it decreased ACSL4 levels in the spinal cord (Kang et al., 2020). Thus, researchers have not clearly determined whether PACs decrease arachidonic acid or adrenic acid levels to inhibit ferroptosis. Hence, this topic requires in-depth investigation. Alox15B is involved in the cellular lipid synthesis process and is needed for ferroptosis. Alox15B was reported to be widely expressed in neutrophils and macrophages (Archambault et al., 2018; Snodgrass and Brüne, 2019). The decrease in Alox15B levels induced by the PAC treatment after SCI suggests that Alox15B is likely a cellular target of PACs since it is extensively expressed in neutrophils and macrophages. Fan et al. (2021) reported that liproxstatin-1 (Lipro-1) is an inhibitor that effectively blocks ferroptosis and elucidated the possible pharmacological effects of Lipro-1 on suppressing ferroptosis and its signaling pathways. First, GPX4 is a critical factor regulating ferroptosis (Yang et al., 2014). In the spinal cord, GPX4 is primarily expressed in neurons and oligodendrocytes, but not in astrocytes (Hu et al., 2019). Interestingly, in contrast to a neuronal cytoplasmic localization, GPX4 localizes to the nucleus in oligodendrocytes *in vivo* (Hu et al., 2019). GPX4 consumes GSH to inhibit lipid peroxidation, particularly lipid hydroperoxidation, in biological membranes (Brigelius-Flohé and Maiorino, 2013). On the one hand, Lipro-1 restored GPX4 to normal levels in ferroptotic oligodendrocytes to inhibit the ferroptosis signal within the nucleus (Imai et al., 2017). On the other hand, Lipro-1 increased GSH levels to enhance the anti-ferroptosis system (Fan et al., 2021). In addition, one existing study reported that Lipro-1 was capable of decreasing mitochondrial ROS levels (Feng et al., 2019). Based on these results, Lipro-1 may inhibit oligodendrocyte ferroptosis. After SCI, damage to oligodendrocytes induced by ferroptosis potentially aggravates demyelination. However, Lipro-1 inhibits oligodendrocyte ferroptosis to repair SCI. In summary, Lipro-1 may be a potential drug for the treatment of SCI.

### The Effect of Ferroptosis on TBI

Spinal cord injury and TBI have similar pathophysiological mechanisms, and ferroptosis occurs in TBI. Xie et al. (2019) revealed that the mRNA levels of genes related to ferroptosis [such as Ribosomal Protein L8(RPL8), Iron Responsive Element Binding Protein 2(IREB2), Prostaglandin-endoperoxide synthase 2 (PTGS2), and ATP5G3] were remarkably increased within the cortex of a controlled cortical impact injury (CCI) mouse model compared to sham animal model groups. Additionally, GPX activity decreased noticeably after 6 h, 1 and 3 days. The malondialdehyde (MDA) concentration and lipid ROS levels increased noticeably at 6 h, peaked on day 3 and returned to baseline on day 7. Mitochondrial morphological characteristics are some of vital factors that support ferroptosis. Meanwhile, using TEM, the authors observed shrunken mitochondria in the neuronal soma 3 days after TBI, thus providing powerful evidence for the appearance of ferroptosis after TBI. As proven in subsequent studies, ferroptosis mainly causes severe secondary injury after TBI, and Ferrostatin-1 reduces iron accumulation, attenuates neuronal degeneration in mouse with TBI and ameliorates TBI-induced motor and cognitive deficits (Wu et al., 2019). As confirmed by the aforementioned results, ferroptosis plays an important role in TBI (Xie et al., 2019). Kenny et al. (2019) added RSL3 (an inhibitor of GPX4) to cultures of HT22 cells and observed increased cell death. The author and colleagues discovered that baicalein effectively attenuated the oxidation of arachidonic/adrenic acid-containing-phosphatidylethanolamine (AA/AdA-PE), a key step in ferroptosis, in a rat CCI model. Moreover, miR-212-5p is likely to prevent ferroptotic neuronal death in a mouse CCI model partially by targeting PTGS2 (Xiao et al., 2019). Xiao et al. (2019) investigated whether miR-212-5p alters ferroptotic neuronal death in mice with TBI and detected ferrous ion and malondialdehyde accumulation (MDA), the expression levels of the ferroptosis-related molecules at 6, 12, 24, 48, and 72 h and significant upregulation of GPX4 and ACSL4 expression was observed at 6 h following in a mouse CCI model. In this study, the authors reported a significant association between ferroptosis and miR-212-5p expression in a mouse CCI model. The possible effect of miR-212-5p on the ferroptosis process was illustrated. The authors assessed the ferroptosis-inducing activity of RSL3 in HT-22 and Neuro-2a cell lines. Then, miR-212-5p overexpression was induced by transfecting HT-22 and Neuro-2a cells with a miR-212-5p mimic and its expression was silenced by transfecting a miR-212-5p inhibitor. Comparison with the cells transfected with the mimic negative control, miR-212-5p overexpression reduced the cell death rate (Xiao et al., 2019). According to the results, miR-212-5p critically controls ferroptosis. Using a dual-luciferase assay, the authors found that PTGS2 may be a target gene with possible miR-212-5p binding sites. PTGS2, also known as cyclooxygenase-2 (COX-2), is a vital enzyme in prostaglandin biosynthesis that simultaneously produces dioxygenase and peroxidase (Amadio et al., 2017). Cyclooxygenases catalyze lipid oxidation, which is involved in ferroptosis. The current studies on miRNA-mediated regulation of ferroptosis still have some deficiencies. First, the cell type-specific expression of miR-212-5p has not been clearly determined. In-depth research should be carried out to determine its expression pattern. Second, further research is needed to determine whether other miRNAs regulate ferroptosis in TBI. To date, few studies have been conducted on this topic. Thus, it is a very promising direction.

## Discussion and Perspectives: Will Ferroptosis Be a Future Direction?

Here, we focus on the effects and therapeutic potential of ferroptosis in a wide range of acute CNS trauma conditions, including TBI and SCI. Although considerable progress has been achieved in the study of ferroptosis, many problems remain to be solved. The pharmacologic effects of molecules inhibit ferroptosis maliy lie at the iron, amino acid and lipid metabolism. Currently, studies on the pharmacologic inhibition of ferroptosis in CNS trauma are limited. Therefore, it’s necessary to found other potential drugs to depress ferroptosis in SCI and TBI. In other neurological diseases, many drugs have been proved to alleviate ferroptosis and promote nerve repair, such as edaravone and *N*-acetylcysteine. It was reported that edaravone inhibited ferroptosis by scavenging chain-initiating water-soluble radicals and chain-carrying lipid peroxyl radicals due to its amphiphilicity, in ischemic stroke and amyotrophic lateral sclerosis (Homma et al., 2019; Spasić et al., 2020). Moreover, it was found that *N*-acetylcysteine reduced neuronal ferroptosis and improved behavior following intracerebral hemorrhage in mice through neutralizing nuclear ALOX5-derived toxic lipid species which is involved in the cellular lipid synthesis process and is needed for ferroptosis (Karuppagounder et al., 2018). However, the effects of above drugs involving ferroptosis regulation have not been investigated in CNS trauma. So whether the drugs can inhibit ferroptosis in TBI and SCI remains to be further studied.

The relationship between ferroptosis and other forms of cell death is unknown. For example, the membrane lipid composition regulates the processes of ferroptosis and necroptosis. In a mouse kidney ischemia-reperfusion injury (IRI) model, Muller et al. observed increased time-dependent sensitivity to necroptosis in ACSL4-knockout cells using CRISPR/Cas9-based genome-editing technology (Müller et al., 2017). Mixed lineage kinase domain-like pseudokinase (MLKL) knockout sensitized cells to ferroptotic death. Thus, necroptosis and ferroptosis are intertwined in murine renal IRI. MLKL drives basal resistance to ferroptosis by depleting PUFAs, and ACSL4 drives basal resistance to necroptosis by making the membrane less susceptible to MLKL-driven membrane permeabilization (Chen Y. et al., 2020). In addition, autophagy is associated with ferroptosis. Some scholars found that heat shock protein 90, an evolutionarily conserved and ubiquitously expressed molecular chaperone, participated in ferroptosis by regulating the levels of Lamp-2a in the chaperone-mediated autophagy pathway (Thayyullathil et al., 2021). Other scholars also found that autophagy degrades ferritin in neurons to increase intracellular free iron levels and then promote the occurrence of ferroptosis in a rat model of subarachnoid hemorrhage (Patil et al., 2021). Moreover, autophagy participates in the downstream execution of ferroptosis (Thayyullathil et al., 2021). According to Ma et al. (2017) ferroptosis and autophagy do not regulate each other, whereas they were triggered by iron-dependent ROS. Indeed, all of these results were obtained from animal models of other diseases. Further studies are needed to explore whether consistent changes occur in CNS trauma and the association of ferroptosis and other forms of cell death.

Given the complexity of CNS trauma, the biochemical regulation and sensitivity of various cell types, such as oligodendrocytes, microglia, astrocytes, or neurons, to ferroptosis also require discussion. Previous studies have confirmed that cell death after CNS trauma is an important cause of the loss of nerve function (Hu et al., 2020; Shi et al., 2021). Different cell types, such as neurons, astrocytes, microglia, and oligodendrocytes, play various roles in CNS trauma. Studies of the sensitivity of different cell types to ferroptosis are crucial. Recent studies may provide some answers. Scholars have detected lower GSH levels in oligodendrocytes than in astrocytes; thus, the GSH/GPX4 system is insufficient to prevent lipid peroxidation (Thorburne and Juurlink, 1996). Additionally, the total iron content in oligodendrocyte precursor cells is much higher than that in astrocytes. Iron is essential for ferroptosis because of its ability to generate ROS *via* redox reactions (Jiang et al., 2021). For these two reasons, oligodendrocytes are more sensitive to ferroptosis. In addition, the expression of GPX4 is downregulated in subjects with SCI and TBI. Thus, oligodendrocytes are likely to undergo ferroptosis in individuals with these diseases, which would subsequently aggravate demyelination. Furthermore, Chen L. et al. (2015) found that GPX4 ablation significantly increased the number of reactive astrocytes, which led to astrogliosis. Their results showed an increased number of astrocytes following the induction of ferroptosis, resulting in scar formation. However, this result is opposite to the phenomenon by which ferroptosis leads to cell death. Thus, further study is needed to explore the sensitivity of astrocytes to ferroptosis. Many experiments have shown that neurons are very sensitive to ferroptosis. Treatment with the ferroptosis inhibitor ferrostatin-1 significantly reduces neuronal cell death (Xie et al., 2019). The role of microglia in ferroptosis has been less well studied. Therefore, these cells will be the focus of subsequent studies.

## Conclusion

Ferroptosis, a novel type of cell death, has recently aroused substantial interest. Studies of ferroptosis have achieved significant progress. The ferroptosis mechanism mainly includes iron toxicity, inactivation of GPX4 and system Xc^–^, ROS accumulation and ultimately lipid peroxidation. Nevertheless, insights into ferroptosis in CNS trauma remain preliminary, and a number of problems remain to be studied.

## Author Contributions

XH and YX searched and reviewed literature and drafted the manuscript and revision. HX and CJ discussed and revised the manuscript. HZ, HS, and YL provided the critical comments. WN and KZ designed and formulated the review theme and revised and finalized the manuscript. All authors contributed to the article and approved the submitted version.

## Conflict of Interest

The authors declare that the research was conducted in the absence of any commercial or financial relationships that could be construed as a potential conflict of interest.

## Publisher’s Note

All claims expressed in this article are solely those of the authors and do not necessarily represent those of their affiliated organizations, or those of the publisher, the editors and the reviewers. Any product that may be evaluated in this article, or claim that may be made by its manufacturer, is not guaranteed or endorsed by the publisher.

## Abbreviations

CNS: Central nervous system
SCI: Spinal cord injury
TBI: Traumatic brain injury
L-ROS: Reactive oxygen species
L-OOH: Lipid hydroperoxides
L-OH: lipid alcohols
GSH: Glutathione
CoA: Coenzyme A
OH: Soluble hydroxyl
RO: Lipid alkoxy
RSL3: (1S,3R)-RSL3
Hb: Hemoglobin
TFR1: Transferrin receptor 1
DMT1: Divalent metal transporter 1
LIP: Labile iron pool
PUFA: Polyunsaturated fatty acid
ACSL4: Acyl-CoA synthetase long-chain family member 4
LPCAT3: Lysophosphatidylcholine acyl transferase 3
PLs: Phospholipids
BSO: Buthionine sulfoximine
GPX4: Glutathione peroxidase 4
DPI2: Diverse pharmacological inhibitor-2
NAPQI: N-acetyl -p-benzoquinone imine
ROOH: Reduce organic peroxide
H_2_O_2_: Free hydrogen peroxide
L- O-: Alkoxy radicals
Tf: Transferrin
TFR1: Transferrin receptor 1
STEAP3: Six-transmembrane protein prostate 3
DMT1: Divalent metal transporter 1
PCBP1: Poly(rC)-binding protein
AA: Catalyses arachidonic acid
AdA: Adrenic acid
PE: Phosphatidylethanolamines
LOXs: Lipoxygenase
15-LOX: 15-lipoxygenase
TEM: Transmission electron microscopy
IREB2: Iron-responsive element-binding protein 2
ACSF2: Acyl-CoA synthetase family member 2
DFO: Deferoxamine
PAC: Proanthocyanidin
ALOX15B: Arachidonate 15-Lipoxygenase Type B
TBARS: Thiobarbituric acid reactive substances
Nrf2: Nuclear factor-erythroid 2-related factor 2
HO-1: Heme oxygenase-1
GSSG: Oxidized glutathione
Lipro-1: Liproxstatin-1
IREB2: Iron Responsive Element Binding Protein 2
PTGS2: Prostaglandin-endoperoxide synthase 2
RPL8: Ribosomal Protein L8
CCI: Cortical impact injury
MDA: Malondialdehyde
AA/AdA-PE: Arachidonic/adrenic acid-containing-phosphatidylethanolamine
COX-2: Cyclooxygenase-2
IRI: Ischemia-reperfusion injury
MLKL: Mixed lineage kinase domain-like pseudokinase.
